# GH/IGF-1 Axis as a Gerotherapeutic Target

**DOI:** 10.1093/geroni/igaf122.1103

**Published:** 2025-12-31

**Authors:** Sofiya Milman

**Affiliations:** Albert Einstein College of Medicine, Albert Einstein College of Medicine, New York, United States

## Abstract

While growth hormone/ insulin like growth factor-1 (GH/IGF-1) is a well-documented modulator of aging and longevity in model organisms, its role in human aging is less established. Growing epidemiologic and genetic evidence from general population and exceptional longevity cohorts is pointing to attenuated GH/IGF-1 signaling as a mediator of healthy aging and protection against age-related diseases, including cancer, diabetes, dementia, and cardiovascular disease. This evidence has led to preclinical trials of newly developed and repurposed therapeutics that diminish GH/IGF-1 signaling, with the potential to delay aging and age-related diseases in people.

